# Revealing biogeochemical signatures of Arctic landscapes with river chemistry

**DOI:** 10.1038/s41598-019-49296-6

**Published:** 2019-09-09

**Authors:** Arial J. Shogren, Jay P. Zarnetske, Benjamin W. Abbott, Frances Iannucci, Rebecca J. Frei, Natasha A. Griffin, William B. Bowden

**Affiliations:** 1 Michigan State University, Department of Earth and Environmental Sciences, East Lansing, Michigan 48824 USA; 2 Brigham Young University, Department of Plant and Wildlife Sciences, Provo, Utah 84602 USA; 3 University of Vermont, Rubenstein School of Environment and Natural Resources, Burlington, Vermont 05405 USA; 4 University of Alberta, Department of Renewable Resources, Edmonton, Alberta T6G 2R3 Canada

**Keywords:** Freshwater ecology, Freshwater ecology, Element cycles, Element cycles

## Abstract

Riverine fluxes of carbon and inorganic nutrients are increasing in virtually all large permafrost-affected rivers, indicating major shifts in Arctic landscapes. However, it is currently difficult to identify what is causing these changes in nutrient processing and flux because most long-term records of Arctic river chemistry are from small, headwater catchments draining <200 km^2^ or from large rivers draining >100,000 km^2^. The interactions of nutrient sources and sinks across these scales are what ultimately control solute flux to the Arctic Ocean. In this context, we performed spatially-distributed sampling of 120 subcatchments nested within three Arctic watersheds spanning alpine, tundra, and glacial-lake landscapes in Alaska. We found that the dominant spatial scales controlling organic carbon and major nutrient concentrations was 3–30 km^2^, indicating a continuum of diffuse and discrete sourcing and processing dynamics. These patterns were consistent seasonally, suggesting that relatively fine-scale landscape patches drive solute generation in this region of the Arctic. These network-scale empirical frameworks could guide and benchmark future Earth system models seeking to represent lateral and longitudinal solute transport in rapidly changing Arctic landscapes.

## Introduction

The fate of carbon and nutrients liberated from rapidly changing Arctic landscapes is a factor of critical concern, affecting both local habitat and global climate^1–5^. Previous studies of the largest Arctic watersheds have revealed increases in circumpolar riverine concentrations and fluxes for nearly all solutes^6–8^, a signal of Arctic landscape change  that is expressed in river hydrochemistry. These observed increases in riverine carbon and nutrient fluxes^9,10^ are likely driven by a combination of discrete and diffuse dynamics. First, spatially discrete permafrost collapse features can rapidly deliver permafrost solutes to surface water networks^3,11,12^. Thermo-erosion (hereafter thermokarst) features often form on the banks of rivers and lakes where water acts as a thermal trigger for permafrost degradation^13–15^. Consequently, thermokarst formation short-circuits soil flow-paths that would remove dissolved organic matter, delivering unprocessed nutrients and carbon from degrading permafrost directly to aquatic ecosystems^3,12,16^. Second, lateral connectivity can increase between terrestrial and aquatic ecosystems via active layer deepening during the hydrologic season^17–22^. Third, Arctic hydrology is changing rapidly, including larger storm pulses that accelerate flow through thawed soils and extended flow seasons that allow leaching of nutrients after cessation of plant growth^23^. Together, thawed soils and increased stream flow can enhance lateral and longitudinal solute flux by effectively reducing the exposure of solutes to removal and retention mechanisms in terrestrial and aquatic environments^22,24,25^. Regardless of the underlying drivers of increased nutrient and carbon inputs, observed and projected changes in Arctic hydrologic regimes and stream solute exports have profound implications for freshwater and coastal ecosystems^26^. As permafrost ecosystems respond to climate change, the availability of permafrost nutrients will regulate key components of the global carbon cycle and energy balance^9,27–33^, including the magnitude of carbon dioxide fertilization of net primary productivity^34–36^, the persistence of soil organic matter^37,38^, vegetation community structure^39,40^, and the net energy balance of the land surface^33^.

**Figure 1:**
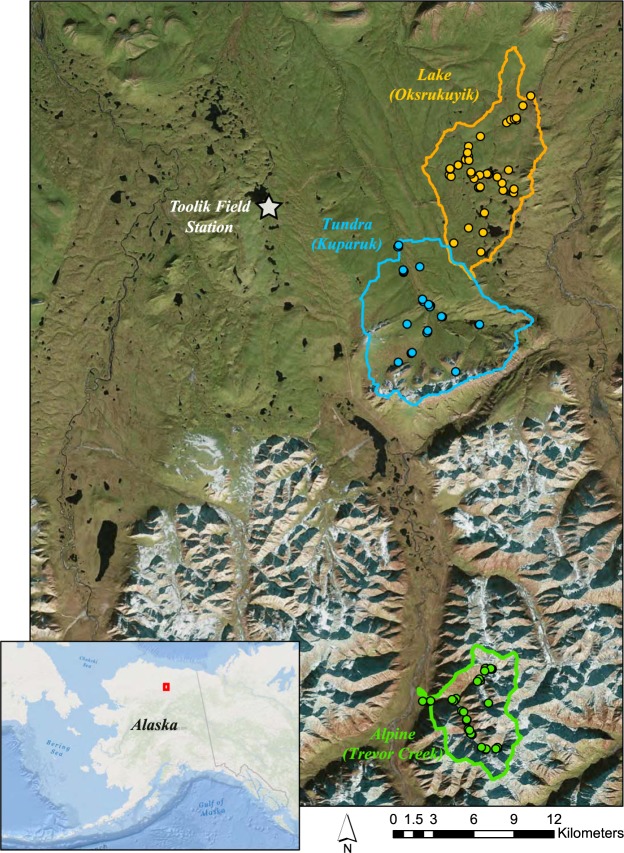
Sampling locations in the three Arctic catchments in Northern Alaska near the Toolik Field Station. We sampled 42 sites in the Tundra (blue), 41 sites in the Lake (orange), and 31 sites in the Alpine (green) watersheds. Map created in ArcGIS Pro (version 2.2.4). Imagery from Esri, DigitalGlobe, Earthstar Geographics, CNES/Airbus DS, GeoEye, USDA FSA, USGS, Aerogrid, IGN, IGP, and the GIS User Community.

To explain how, where, and why changes in lateral solute flux are occurring in Arctic landscapes, we must identify the drivers of this signature, and quantify the patch size or spatial scale of sources and sinks of dissolved carbon and nutrients^9,37,41–45^. However, the mismatch between scales of observations and the scales driving ecosystem processes complicates attribution of landscape factors driving hydrochemical flux^46^. Most measurements of Arctic river chemistry are from the outlets of large rivers (>100,000 km^2^), where observations cannot distinguish the relative importance of diffuse (e.g., vegetation community change or active-layer deepening) and discrete (e.g., abrupt permafrost collapse or fire) solute release mechanisms^47^. Alternatively, experimental and process-based modeling studies have characterized nutrient processes at the much smaller plot-scale (<1–100 m^2^) in terrestrial environments^48,49^ or the reach-scale (~100–200 m) in lotic environments^50–52^. While environmental factors at these disparate spatial scales are only rarely connected, a spatially-extensive, synoptic sampling framework may reveal the spatial scales driving terrestrial nutrient export and river hydrochemistry^53^. In this context, we collected and analyzed a synoptic dataset from 120 nested subcatchments within three ecologically distinct watersheds on the North Slope of Alaska. Our main goals were to understand material linkages between terrestrial and aquatic ecosystems and to refine predictions of material budgets under present and future climate-change scenarios. While we measured a wide suite of biogeochemical solutes, we focus primarily on nitrogen, phosphorus, and carbon species, which regulate and reflect fundamental ecosystem processes in Arctic freshwater ecosystems. These solutes are also of central concern in regional measurements and models of permafrost ecosystems^5^. We collected samples early (first week of June) and late (last week of August) in the flow season, to capture conditions across the range of soil thaw depths. We performed each sampling campaign at relatively stable flow conditions (SI Fig. 2). For each watershed, we derived three metrics to characterize Arctic hydrochemistry^47^: *variance collapse*, *subcatchment leverage*, and *spatial stability* for dissolved carbon, nitrogen, and phosphorus. Variance collapse provides information about the patch size of major source and sink processes that contribute to watershed hydrochemical fluxes^47^. Subcatchment leverage quantifies specific areas that produce or remove major solutes^47^. Finally, spatial stability indicates if solute sourcing and transport dynamics are stable and can be represented with temporally-sparse sampling^47^. We hypothesized that these metrics of hydrological and biogeochemical processes would be organized by the local and regional watershed characteristics. These controlling factors include hillslope connectivity and slope^54–56^, terrestrial vegetation type and productivity^57^, and stream-lake connectivity^58,59^, in addition to the presence of permafrost and the depth of the seasonally thawed “active layer”^60^. Consequently, we chose study catchments that differed across these gradients, including a low-productivity and high-gradient alpine catchment (Alpine); a high-productivity, low-gradient tundra river (Tundra); and a lake-dominated, low-gradient tundra river (Lake) (Fig. 1, SI Table 1).

## Spatial Extent of Ecosystem Processes Driving Lateral Nutrient Flux

**Figure 2:**
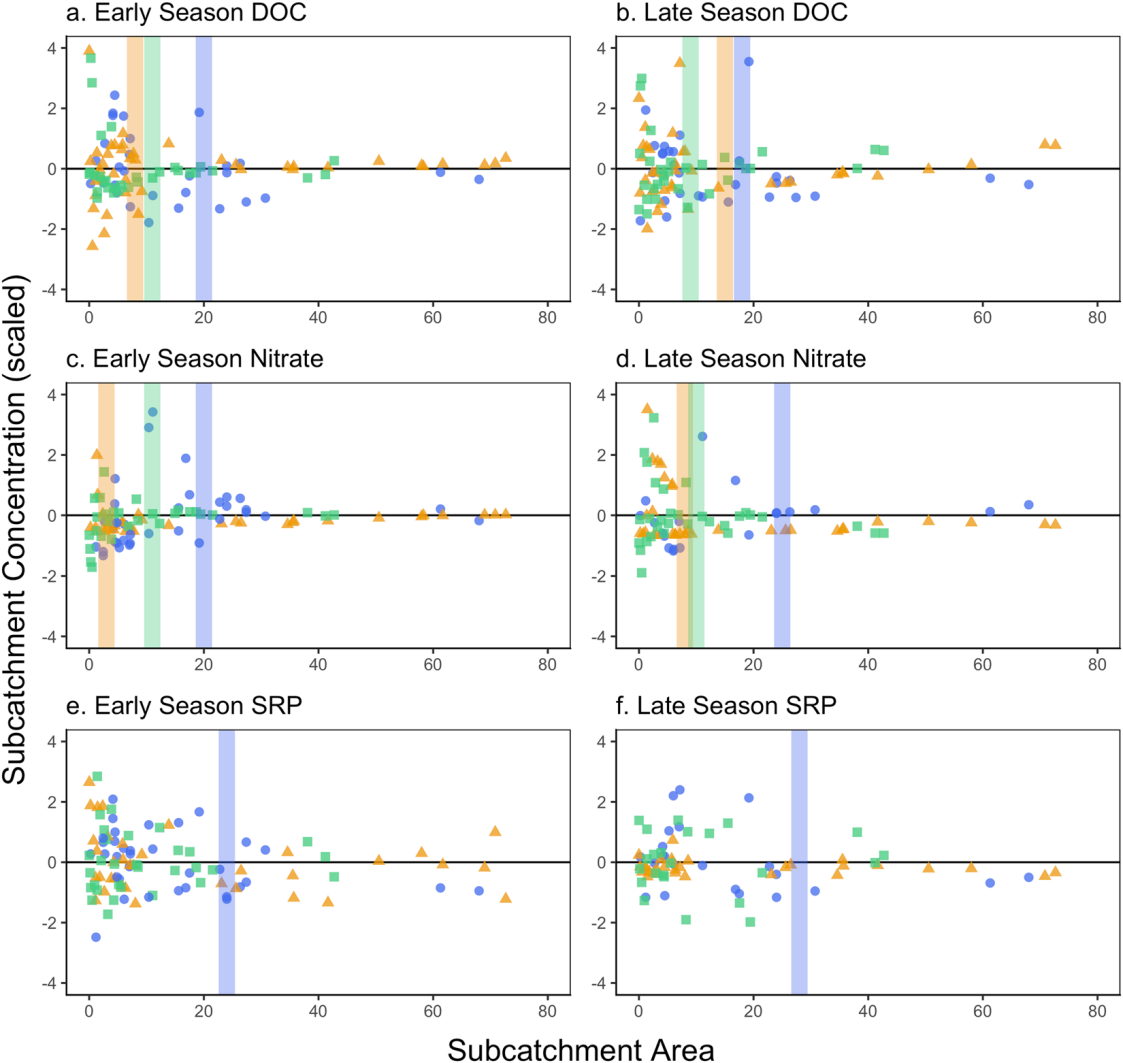
Variability in concentration for subcatchments of differing sizes in Tundra (blue circles), Lake (orange triangles), and Alpine (green squares) for Early and Late season. Points represent scaled mean values for (**a**) dissolved organic carbon (DOC), (**b**) nitrate (NO_3_^−^) and (**c**) soluble reactive phosphorus (SRP). The colored vertical lines represent statistical changes in spatial variance among subcatchments based on the change point analysis implemented for each watershed. Concentrations were scaled by subtracting the mean and dividing by the standard deviation to facilitate comparison of changes in variance.

We assessed the spatial and temporal patterns in solute processing within each watershed using a synoptic sampling approach, which allowed us to quantify variance collapse, subcatchment leverage, and spatial stability (Fig. 2). At any moment in time, the spatial extent and distribution of nutrient sources and sinks in the landscape can be assessed by the spatial scale of the variance collapse for each solute among reaches of the watershed^47^. Stated differently, the point where the variance of solute concentration collapses close to zero provides a watershed area ideal for understanding solute source and sink patterns. Broadly, we expected that each watershed would have unique and defined areas of variance collapse for dissolved organic carbon (DOC), nitrate (NO_3_^−^), and soluble reactive phosphorus (SRP), indicative of the size of landscape sources and sinks in that watershed. Alternatively, if no variance collapse threshold is observed, this indicates that the spatial drivers are larger than the watershed or in-stream processes and obscure terrestrial signals. Below, we outline our results for each solute.

The variance in DOC and NO_3_^−^ collapsed in all watersheds in both early and late seasons, though variance thresholds reflecting patch size differed by watershed and season (Fig. 2a,b). The unique variance collapse values for DOC and NO_3_^−^ in the Lake, Tundra, and Alpine watersheds suggest distinct landscape and surface-water network drivers generating and removing these constituents^46^. The Tundra watershed, for example, had relatively large-scale patches for DOC (18–20 km^2^) and NO_3_^−^ (20–21 km^2^), which we interpret to be the result of relatively homogenous land cover and topography. This homogeneity may reduce small-scale variation and reveal larger patches potentially associated with surficial geology and glacial history. For the Tundra watershed, there was likely a strong network constraint on solute spatial variability, creating similar spatial scales through time and for all three solutes. In contrast, variance collapse thresholds for NO_3_^−^ in the Lake and Alpine watersheds occurred at small to intermediate scales (3–15 km^2^), reflecting finer-grained heterogeneity of nutrient sources and sinks^46^. Stated differently, both Lake and Alpine watersheds had high variability in DOC and NO_3_^−^ concentrations in the smaller headwater catchments, with signals quickly reduced as surface-water networks mixed these small patches as a result of stream-lake interactions^61,62^ or network topography^54,63^. For all watersheds, we did not find substantial changes in DOC or NO_3_^−^ variance collapse (>5 km^2^) across seasons, implying that stream network topography, and not seasonality, largely determined observed spatial trends. Overall, the DOC and NO_3_^−^ variance collapse scales confirm that the watersheds themselves are distinct, both in terms of the patch scale of apparent drivers that contribute to solute and carbon export, and landscape-driven network constraints. Together, these results reveal the importance of intermediate landscape scales between 3–30 km^2^ as regulators of Arctic carbon and nutrient sources and sinks, and the utility of synoptic campaigns for identifying emergent watershed patterns. However, variance collapse thresholds for SRP (Fig. 2c,f) were less clear. We found a statistically significant variance collapse for the Tundra watershed (~28 km^2^), but for the Lake and Alpine watersheds, we found no significant variance collapse for SRP. This lack of changes in variation with increased spatial scale could be due to rapid  in-stream processing, which erases the terrestrial delivery signal. Phosphorus is highly limiting in stream and lake ecosystems on the North Slope (^64–66^), meaning that phosphorus concentration at a particular moment in time in a stream network could be primarily a consequence of immobilization and mineralization in the aquatic environment^67^.

## Seasonal Changes in River Network Leverage Indicate Strong Topographic Controls

**Figure 3:**
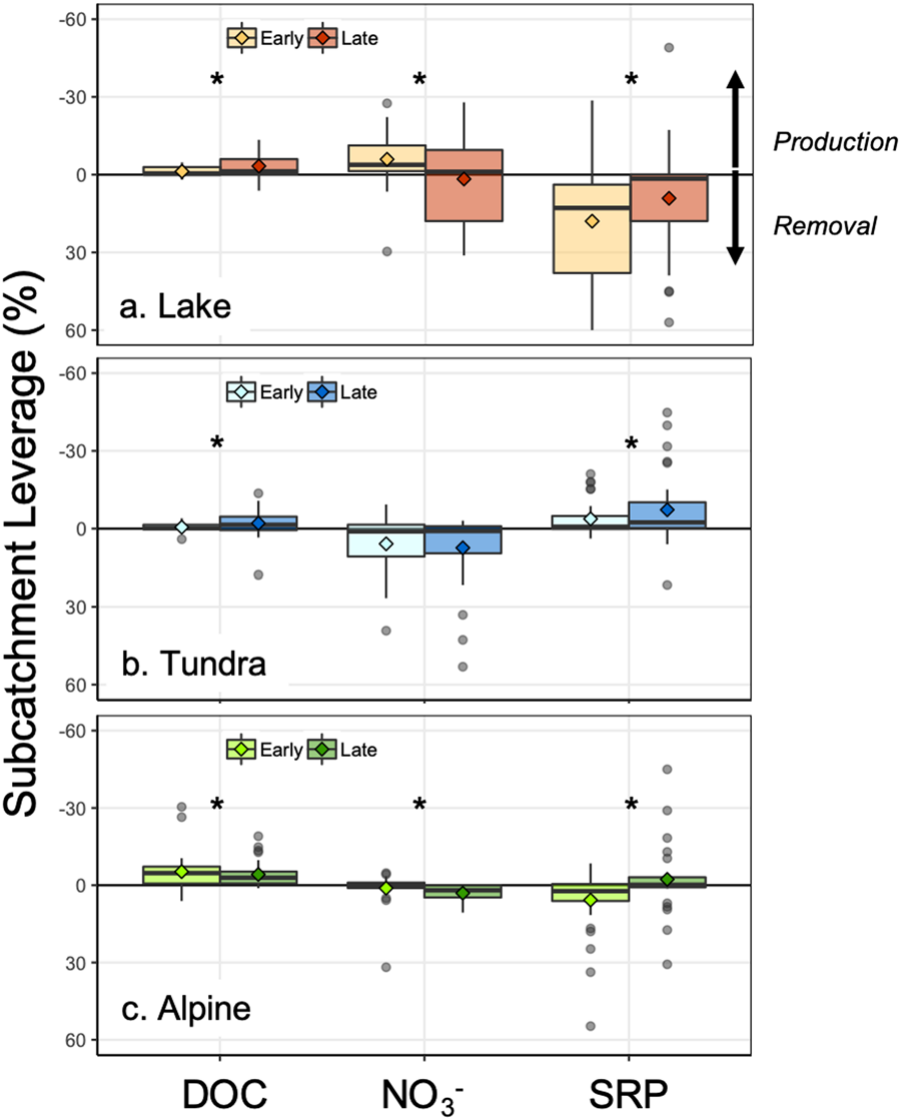
Early and late season mean subcatchment leverage across all sampling sites for each watershed. (**a**) Lake (orange bars), (**b**) Tundra (blue bars), and (**c**) Alpine (green bars). Note that axes have been reversed, with negative leverage (<0) values indicating net production, positive values (>0) suggesting net removal, and values near 0 implying net near conservative leverage. Diamonds show the mean, and boxplots show the median, quartiles, 1.5 times the interquartile range, and points beyond 1.5 times the IQR.

We quantified the influence or leverage of each subcatchment on nutrient export by multiplying the subcatchment concentration with the subcatchment area standardized to the watershed outlet (Fig. 3). If the mass balance of a solute in a watershed is neutral (i.e. conservative mixing with no net production or removal), leverage values will have a mean of zero^47^. If there is more solute in the headwaters of a watershed than can be accounted for at the outlet, the mean leverage will be positive, representing in-network uptake, and conversely negative mean leverage represents in-network production. This analysis thus quantifies the net watershed retention or release and also the spatial location of that reactivity, allowing us to assess the importance of influential ecosystem control points^68^ in terrestrial and aquatic environments. We note that comparing the mean and distribution of leverage values reflects net ecosystem behavior and not the driving mechanisms, and  therefore represents the combination of many potential biological and physical removal and production processes^4,69–71^. We expected that each watershed would have solute-specific and season-dependent subcatchment leverage values.

**Figure 4:**
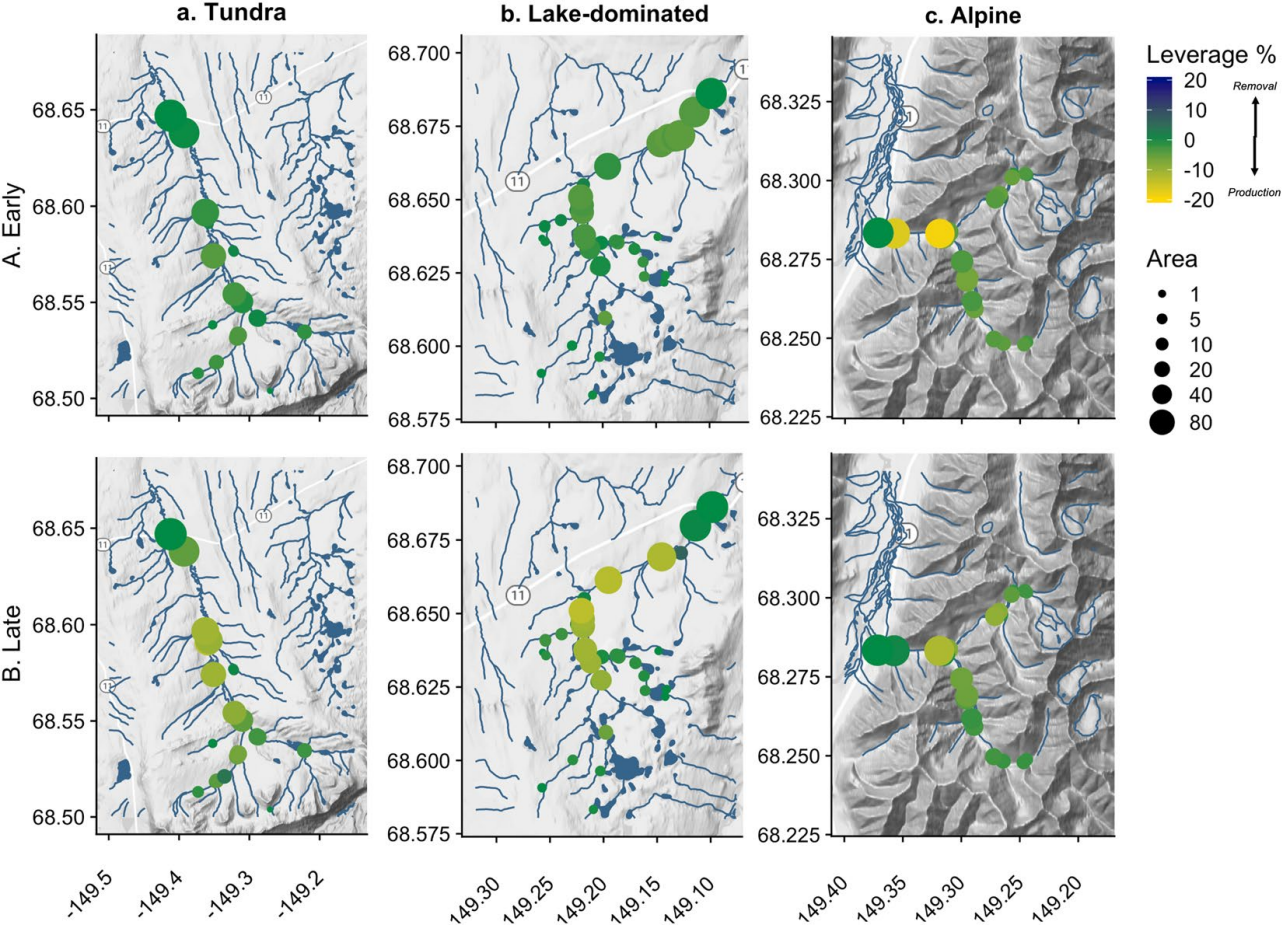
Normalized concentration (subcatchment leverage) mapped for DOC from (A) Early to (B) Late season in the (**a**) Tundra, (**b**) Lake, and (**c**) Alpine watersheds. Rivers and lakes are shown in blue. Point size indicates total subcatchment drainage area at each sampling location; smaller points indicate smaller tributaries, while the largest show main-stem or larger tributary sites. Color scale indicates leverage percent, with blue indicating lower concentrations relative to the watershed outflow and yellow noting higher concentrations than at the outflow.

Across all watersheds, we expected that DOC leverage would consistently indicate net production. In the Arctic, DOC is abundant, and lateral terrestrial and riparian zones are a large, readily available carbon pool^72–74^, though its biological reactivity is often low despite rapid instream processing (e.g., photo and biodegradation^75^). In our study watersheds, we found mean DOC leverage values that were always negative, but generally lower in magnitude than SRP and NO_3_^−^ (leverage <5%), indicating consistent but low net DOC production throughout the stream network (Fig. 3a). Accordingly, we observed relatively dispersed, DOC production throughout all three watershed networks (e.g., Fig. 4). However, while production was consistent, the magnitude of net DOC production changed seasonally within each watershed (Fig. 3). For example, net DOC production was higher in the late season than the early season in the Lake and Tundra watersheds. Although this seasonal difference was consistent for these two watersheds, we suspect that they experience different underlying mechanisms. In the Tundra watershed, DOC is primarily terrestrially-derived (allochthonous), with increases in DOC production a result of enhanced hillslope connectivity^76–78^. In the Lake watershed, early-season DOC is likely primarily comprised of allochthonous inputs from the spring freshet, but late-season DOC may become more internally-derived (autochthonous)^79^. Further, lakes may be subsidized by stream-derived nutrients (e.g., SRP^80^), altering lake nutrient stoichiometry and increasing organic matter processing rates^71^. In contrast, mean DOC leverage approached 0 in the Alpine watershed (Fig. 3a,b, summary statistics in SI Table 2). In the Alpine catchment, low net leverage across seasons reflects conservative transport throughout the steep subcatchments, as a result of rapid hydrologic flushing and contact with mineral horizons poor in organic matter^56,77^. However, this finding is contrary to the expectation that DOC production is predicted to increase from early to late season in high-latitude rivers^81^, emphasizing the regional and site variation in DOC production response in the permafrost zone^9^. Overall, our observations underscore that scaling solute behavior across varying landscapes will not be well-captured using a “one-catchment-fits-all” approach.

**Figure 5:**
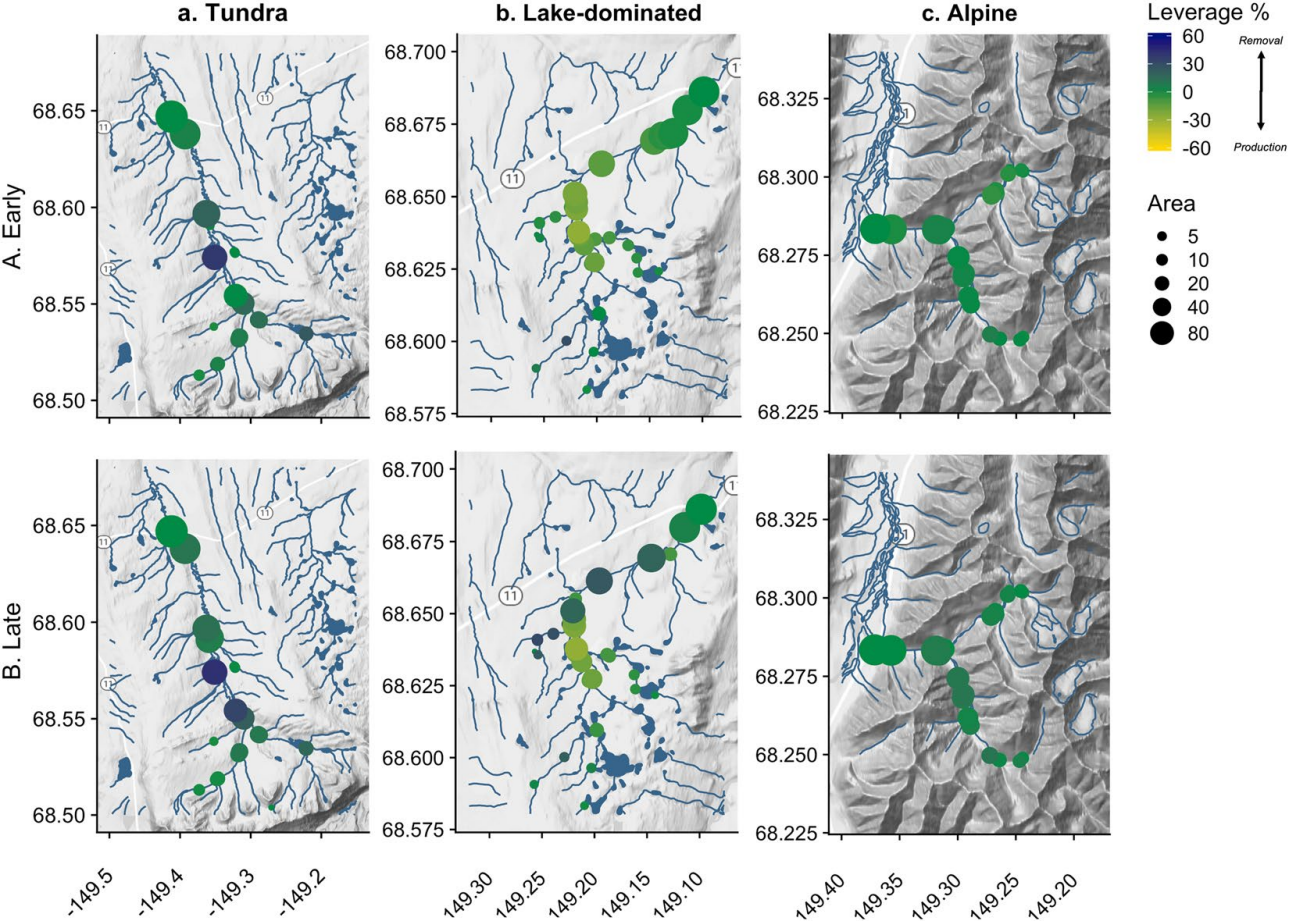
Normalized concentration (subcatchment leverage) mapped for nitrate from (A) Early to (B) Late season in the (**a**) Tundra, (**b**) Lake, and (**c**) Alpine watersheds. Rivers and lakes are shown in blue. Point size indicates total subcatchment drainage area at each sampling location; smaller points indicate smaller tributaries, while the largest show main-stem or larger tributary sites. Color scale indicates leverage percent, with blue indicating lower concentrations relative to the watershed outflow and yellow noting higher concentrations than at the outflow.

Seasonal patterns for NO_3_^−^ were more variable than DOC across watersheds and seasons, revealing the strong control of stream network topography on biogeochemical signals. In the Tundra watershed, mean NO_3_^−^ leverage suggested net removal across early and late seasons, reflecting fairly high biological demand for dissolved nitrogen^82^ (Fig. 3). The location of these influential points for NO_3_^−^ production and removal was relatively consistent through the year (Fig. 5). For example, a single Tundra sampling point along the mainstem maintained high leverage at both sampling points (~38%) indicating net removal processes (Fig. 5a), which is possibly a localized discrete point or reach-scale effect. This influential location in the Tundra river network seems to be an ecosystem control point^68^, and now identified, could be selected as a site location for further observation and experimentation to identify specific mechanisms driving its influence on the watershed network. For example, we expect that this control point arose as a result of a recently formed thermokarst, which increased instream concentrations of NO_3_^−^ and stimulated N demand^3^. In the Lake watershed, mean leverage was seasonably variable, transitioning from net production to net removal. This is likely the result of spatial variability, show in Fig. 5. Here, there is evidence of net production of NO_3_^−^ in early season along the main-stem of the river (larger circles). In contrast, the late season was more spatially variable, with evidence of both NO_3_^−^ production and removal occurring throughout the watershed (variably sized circles). This could be due to the prevalence of lakes, which can reset hydrochemical signals and longitudinal patterns as water moves from between land, lake, and stream within the surface water network^83^. In other words, the material produced or removed in headwater subcatchments is subject to processing and storage within hydrologically-connected lakes^61,62^ before being exported downstream towards the catchment outlet. In contrast to the Tundra and Lake watersheds, both the mean (Fig. 3c) and spatially-distributed (Fig. 5c) estimates both show the prevalence of conservative NO_3_^−^ transport in the Alpine watershed, likely the result of high slope and lower biotic demand for inorganic N^54^.

Like NO_3_^−^, overall seasonal trends in SRP concentration and leverage were watershed-dependent (Fig. 3 and SI Fig. 3), but provide an additional example of how a repeated synoptic approach can identify spatially-discrete influences (e.g., thermokarst inputs^13–15^ or rapid removal^79^) on Arctic watershed biogeochemistry. For example, in the Tundra watershed we found a signal for net SRP production, with increasing production later in the season (Fig. 3). The observation of net SRP production was surprising given that phosphorous is highly limiting in the Arctic generally and this watershed specifically^65^. However, phosphorus release could be the result of discrete sources on the landscape, potentially from thermokarst features observed during our sampling events in the Tundra watershed^84,85^ (SI Fig. 3). Thermokarst features, especially newly formed ones, can be a direct and significant source of SRP to the landscape^3,86^. Indirectly, thermokarst can generate large quantities of labile organic matter that is rapidly mineralized upon thaw^86,87^, which may also liberate a large phosphorus supply in these watersheds. Concurrently, the release of inorganic nutrients could stimulate local productivity^88^, serving as a small patch-scale carbon sink and nutrient retention mechanism^89^. In contrast, in the Lake watershed, stream-to-lake connectivity may have driven a relationship between high biological demand and low overall availability for phosphorus^79,90^. Concentrations throughout the Lake watershed were often low, such that when SRP was available, it was likely  rapidly removed. This is further evidenced by the variance in mean subcatchment leverage, with a range of both net removal and conservative behavior of SRP (Fig. 3), and the spatially-distributed leverage indicative of removal throughout the watershed (SI Fig. 3). Similarly, in the Alpine watershed, SRP production was high in several specific sampling locations for both seasons, though the range of SRP behavior indicated both production and removal points (SI Fig. 3).

While both the whole-watershed and spatially-distributed leverage estimates are based on capturing a limited number of early and late seasons, we suggest that establishing long-term leverage estimates, thus identifying the location and variability of high-leverage sources and sinks^68^, will become increasingly important as a way to track and compare the size and location of nutrient and carbon source over time as the Arctic landscape changes. Despite the expected interaction between Arctic permafrost hydrology and the observed increase of solute export, there is currently no consensus on the magnitude or direction of lateral carbon or nutrient flux from the permafrost zone^9^. As permafrost degradation progresses in high-latitude regions, the relative change in the direction and magnitude of net biogeochemical production and removal effects will ultimately constrain the net carbon and nutrient balance of Arctic ecosystems^43,91^, underscoring the need for further monitoring of these and other vulnerable watersheds.

## Stability of Spatial Patterns of Water Chemistry

**Figure 6:**
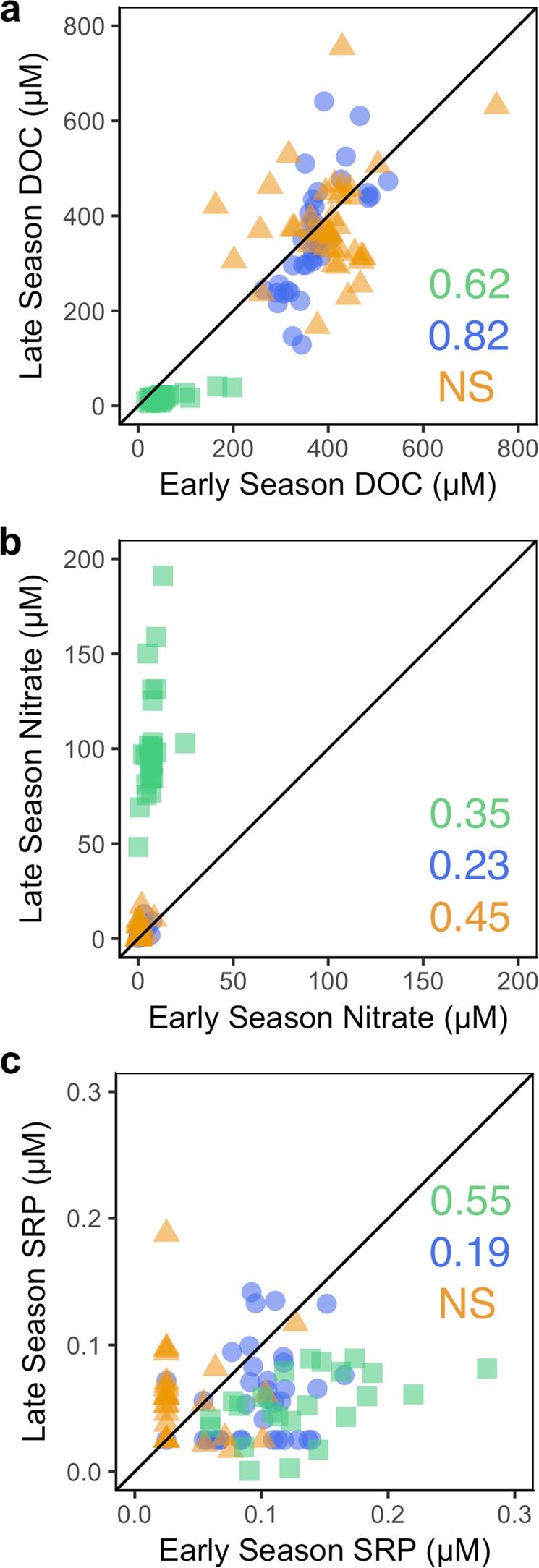
Seasonal stability of reactive solutes for our three Arctic catchments, Tundra (blue circles), Lake (orange triangles), and Alpine (green squares) for early (x-axis) and late season (y-axis). Reactive solutes include. (**a**) DOC, (**b**) NO_3_^−^, (**c**) SRP. Significant rank correlations (a = 0.05) are reported within each panel, and non-significant relationships are denoted by “NS”. Points falling above the 1:1 line increased from early to late season, while points falling below the line decreased from early to late season.

The metric of spatial stability indicates the temporal persistence of spatial patterns of solute concentrations. This metric differs from the previous section where we focused on the persistence of patterns in leverage or influence. Here, we focus on whether patches behave consistently as sources and sinks across time or if they change in function through time, rearranging the spatial configuration of chemistry through the landscape. In our study, spatial stability differed strongly among watersheds and solutes (Fig. 6, SI Table 3), demonstrating temporally variable drivers of DOC, NO_3_^−^, and SRP dynamics. In terms of DOC, we predicted that more homogenous landscapes (e.g., Tundra and Alpine) would be more hydrochemically stable than heterogeneous landscapes (e.g., Lake). Indeed, the Lake watershed underwent a major seasonal reorganization of DOC, with nearly all the spatial structure from the early season rearranged by the end of the year (r_s_ = 0.12, p = 0.27). This reworking at the landscape scale demonstrates the influential nature of lakes in regulating residence time and associated biogeochemistry and longitudinal connectivity^92^, potentially complicating their representation in landscape carbon flux models. We attribute the marked spatial instability for DOC to internal lake productivity and hydrology (e.g. stratification and turnover^93^), which modulates the downstream carbon concentration. This instability contrasts with the highly stable spatial patterns of DOC observed in the similarly vegetated but less lake-influenced Tundra watershed (r_s_ = 0.82) and less-vegetated, lake-absent Alpine (r_s_ = 0.61) watershed (Fig. 6a). In contrast to DOC, NO_3_^−^ patterns indicated major spatial reorganization between early and late season in the Tundra (r_s_ = 0.23), Alpine (r_s_ = 0.45), and Lake watersheds (r_s_ = 0.35) (Fig. 6b). Similarly, patterns for SRP indicated a spatial reorganization in most watersheds, with the highest observed stability in the Lake watershed (r_s_ = 0.55, Fig. 6c), which could be due to increasing mineralization along deeper and longer hillslope and river corridor flow-paths in August^20,94–96^.

While further investigation is needed to determine spatial stability on annual time scales, our finding of watershed-dependent spatial reorganization implies that sampling one subcatchment location repeatedly, as is often done in remote Arctic watersheds, may not capture the underlying seasonal dynamics in the upstream source areas that generate the downstream signal for solutes. Rather than persisting in space, patch source or sink behavior appears to shift seasonally, underscoring the advantages of adding repeated, spatially extensive sampling routines to monitoring efforts.

## The Value of Long-Term Synoptic Datasets

All studies of complex ecosystems involve a tradeoff between sampling frequency and spatial extent. This tradeoff is more pronounced in remote settings, such as the Arctic, where access is limited in space and time. In response to this challenge, many approaches have been proposed to identify ecological patterns and establish underlying terrestrial and aquatic mechanisms. On one hand, using temporally-intensive (i.e. regular or high-frequency) sampling at an Arctic watershed outlet has logistical advantages for access and the use of measurement technology, and has revealed widespread changes in permafrost-zone hydrochemistry^4,6,8,97,98^. On the other, because temporal and spatial variation in watershed exports are tightly interconnected^99^, this variability introduces complexity that more traditional single-location, high-frequency monitoring methods simply cannot capture.

As we have shown with this study, spatially-extensive sampling, while logistically challenging in remote Arctic catchments^53^ and across varying flow regimes, holds significant potential to identify the spatial and temporal scales of dominant drivers of stream hydrochemistry. First, considering the lack of data collected across intermediate spatial scales in river networks of the Arctic, there is very limited spatial benchmarking data for landscape biogeochemical and earth system modeling efforts^100,101^. We argue that synoptic approaches combined with watershed metrics discussed in this work would allow documenting and assessing how Arctic freshwater ecosystems respond to short-term and long-term hydrological and climate-related change. Our approach offers a bridge between studies performed at disparate spatial scales by identifying and quantifying the scale, magnitude, and spatial persistence of key hydrochemical fluxes across spatial and temporal scales, thus providing an opportunity to infer ecosystem functioning at multiple scales^47,99^. Specifically, we demonstrate that most source-sink process controls for DOC, NO_3_^−^, and SRP occur at scales of 3 to 25 km^2^, spatial scales much larger than most current experimental studies, yet much smaller than studies quantifying nutrient export at the outlet of large Arctic rivers. As an additional benefit, when used in compliment to traditional monitoring efforts, synoptic sampling can inform how sensitive watersheds are to temporal variability, such as change in flow and seasonality^47^.

Further, we stress that while the data and discussion presented in this work is Arctic-specific, our method is applicable for watersheds beyond the North Slope of Alaska: repeated synoptic sampling offers a sensitive tool to detect terrestrial and aquatic change across a range of watersheds. Periodic, repeated synoptic sampling and quantification of concentration variance collapse, subcatchment leverage, and spatial stability are beneficial tools for monitoring sensitive subcatchments undergoing systematic change in a way that is entirely complementary to continuous monitoring at a single location^99^. We recognize that studies of watershed hydrochemistry always involve necessary logistic and financial tradeoffs between sampling density, frequency, and extent^102^. However, when possible, we encourage the incorporation of synoptic datasets into river monitoring efforts because they can reveal where and when landscape biogeochemical drivers are changing.

## Supplementary information

Shogren et al. Supplemental Information

## Data Availability

Data for this manuscript are available upon request.
