# Supplementary material for: Revealing biogeochemical signatures of Arctic landscapes with river chemistry
Source: Sci Rep. 2019 Sep 9;9:12894. doi: 10.1038/s41598-019-49296-6 (PMC6733942; doi:10.1038/s41598-019-49296-6)
Supplement: Supplementary file 1 — Shogren et al. Supplemental Information [file 41598_2019_49296_MOESM1_ESM.pdf]

**SUPPLEMENTAL MATERIAL**

**Revealing biogeochemical signatures of Arctic landscapes with river chemistry**

Arial J. Shogren, Jay P. Zarnetske, Benjamin W. Abbott, Frances Iannucci, Rebecca J. Frei, Natasha A. Griffin, William B. Bowden

## Methods

### *Site description and synoptic surveys*

We collected data from three Arctic headwater watersheds on the North Slope of Alaska (Figure 1) that spanned dominant circumarctic vegetation types, permafrost characteristics, and hydrologic conditions: the Kuparuk (hereafter Tundra), Oksrukuyik (Lake), and Trevor Creek (Alpine) (SI Table 1, Figure 1). All three are underlain by continuous permafrost [1]. Both the Kuparuk and Oksrukuyik Rivers drain wet acidic tundra, though the primary difference between the two watersheds is the presence of lakes in the Oksrukuyik watershed basin, and their absence in the Kuparuk (Figure 1). The dominant vegetation across the tundra watershed generally consists of tussock tundra, shrubs, wet sedge, heath, and lichens [1]. Trevor Creek is a higher-elevation alpine watershed (SI Table 1). Climactically, all three watersheds are dominated by a lengthy cold season where snow covers the surface for most of the year. Snowmelt generally occurs between mid-May and mid-June, and the thaw season lasts until mid-September, though snowpack remains until mid-season in Trevor Creek. We sampled each stream network twice (June 5, 2017, and August 27, 2017), where June and August correspond with early and late thaw season, respectively.

### *Solute Analysis*

We syringe-filtered water samples through 25 mm 0.7 µm GF/F filters (Whatman, 0.7 um nominal pore size) into pre-rinsed dark (DOC) and opaque (all other solutes) 60 mL Nalgene HDPE bottles. After filtration, samples were frozen (-4°C) until analysis, with the exception of aliquots for DOC (stored at 2°C until analysis). Prior to refrigeration, DOC samples were acidified with hydrochloric acid to a final normality of 0.01N and refrigerated. We measured $NO_2^-$+$NO_3^-$ on a Lachat QuikChem FIA+ 8000 Series autoanalyzer (QuikChem Method 31-107-04-1-E; detection limit = 0.03 µM). We did not subtract $NO_2^-$ from these results, as concentrations are typically low to undetectable in these systems. SRP was measured on a Shimadzu UV-2600 spectrophotometer (method adapted from Parsons et al. 1984; detection limit = 0.05 µM). DOC was measured on a Shimadzu TOC-L CSH total organic carbon analyzer (combustion catalytic oxidation method; detection limit = 5.0 µM). When values were at or below the limit of detection (LOD), we treated values as a constant value of the LOD divided by 2 for analysis [2].

During each synoptic campaign, we sampled stream water for chemical analysis and physiochemical variables. We collected grab samples of water from each field site in acid-washed 1L amber PCTE bottles. We then lab-filtered aliquots of the sample for specific analytes within 8 hours of collection following LTER protocols.

***Calculating subcatchment metrics: variance collapse, leverage, and spatial stability***

To assess the patch size of solute sources and sinks we determined the threshold of *variance collapse* for each solute from each synoptic sampling event. We ordered the subcatchments by watershed area and used the pruned exact linear time (PELT) method to identify significant increases or decreases in variance using the 'changepoint' package in R [3,4].

Next, we estimated *subcatchment leverage* from the synoptic sampling events. Subcatchment leverage is calculated as: *Subcatchment Leverage* = $[(C_s - C_o) * A_s/A_o * q]$ where $C_s$ and $C_o$ are the concentrations at the subcatchment and whole watershed outlet levels, $A_s$ and $A_o$ are the subcatchment and whole watershed areas, and $q$ is the relative specific discharge at the subcatchment sampling location; i.e., $q = Q_s/Q_o$ where $Q_s$ and $Q_o$ are the area-specific discharges at the subcatchment and whole watershed outlet levels. The Subcatchment Leverage equation simplifies if $Q_s$ and $Q_o$ are similar, i.e., $q \approx 1$. We normalized all concentrations by subtracting the whole watershed mean and dividing by the standard deviation of measured concentrations. Normalized concentrations facilitate comparison of changes in variance over time and among watersheds and ensures that all watersheds converge to the watershed mean. The mean and distribution of subcatchment leverage values can indicate net removal (positive mean leverage) or net production (negative mean leverage) at the watershed scale [4]. In addition to estimating mean subcatchment leverage, we mapped leverage estimated at each sampling point using 'ggmaps' in R [5] and spatial data available through the Toolik Field Station GIS and Remote Sensing center (https://toolik.alaska.edu/gis/data/).

Lastly, we analyzed this data to quantify the *spatial stability* of stream nutrient concentrations and to determine the level of subgrid resolution necessary to represent controls on lateral nutrient loss. Spatial stability is calculated as the standard Spearman's rank correlation coefficient ($r_S$) between instantaneous measurements and varies from -1 to +1. High values of spatial stability (value near 1) means that a single sampling campaign predicts the concentration taken at the next sampling campaign; conversely, a lower value indicates a mismatch between sampling dates and thus a spatial reorganization of stream hydrochemistry. We calculated spatial stability using the correlation function in R (Version 3.3.0) [4].

***Statistical analysis***

We used a linear mixed effects model with site as a random effect to explore landscape predictors (e.g. slope, surface roughness) of surface water concentrations in each watershed using the package 'lme' in R [6]. Finally, we compared both mean solute concentrations and subcatchment leverage estimates between early and late season and among watersheds using 2-way analysis of variance (ANOVA). When the ANOVA was significant, we performed a Tukey's Honesty Significant Differences (HSD) test to compare seasons among watersheds.

SI Table 1 Catchment characteristics June and August 2017, from the Environmental Data Center at Toolik Field Station.

| Catchment | Alpine | Tundra | Lake |
|---|---|---|---|
| Early Sampling Date | June 7 | June 5 | June 3 |
| Late Sampling Date | August 31 | August 27 | August 24 |
| Total Area [$km^2$] | 42.7 | 90.6 | 72.7 |
| Mean Slope [degree] | 29.5 | 3.1 | 3.4 |
| Slope Standard Deviation | 9.4 | 3.1 | 3.7 |
| Mean Roughness | 0.5 | 0.4 | 0.5 |
| Roughness Standard Deviation | 0.05 | 0.02 | 0.1 |
| June Mean Normalized Difference Vegetation Index (NDVI) | 1058 | 4324 | 4789 |
| June NDVI Standard Deviation | 1459 | 522 | 1106 |
| August Mean NDVI | 1172 | 5241 | 4535 |
| August NDVI Standard Deviation | 1540 | 378 | 1022 |
| Surficial Vegetation | Alpine | Tundra | Tundra |
| Surficial Geology | Sagavanirktok Young Glaciated Valleys | Sagavanirktok Old Glaciated Uplands | Sagavanirktok Young Glaciated Uplands |

SI Table 2: Summary statistics for linear models for subcatchment concentrations among catchments, season, and topography (i.e., slope). Asterisks indicate parameter significance at $p < 0.05$ (*), $p < 0.01$ (**), and $p < 0.001$ (***), while (-) denotes no significance. Tukey's HSD for watershed differences are also reported HSD ($p < 0.05$).

| | ***Variable*** | | | | | |
|---|---|---|---|---|---|---|
| **Solute Concentration** | **Catchment Differences** | **Season** | **Slope** | **Site (Random Effect)** | **Whole Model $R^2$** | **Whole Model p** |
| DOC | ** (Alpine < Lake < Tundra) | * | *** | | 0.90 | <0.001 |
| $NO_3^-$ | ** (Tundra = Lake < Alpine) | *** | - | | 0.58 | <0.001 |
| SRP | * (Tundra = Lake < Alpine) | * | - | | 0.08 | 0.03 |

SI Table 3: A) Mean watershed concentrations (μM) of major solutes ± standard error across seasons. B) Early and late season variance collapse thresholds for major solutes. Significant variance collapse (- symbol) was not observed for SRP for Lake and Alpine due to lack of detectable variance collapse. C) and D) Location and direction of subcatchment leverage and spatial stability numbers.

| | **Alpine** | | **Tundra** | | **Lake** | |
|---|---|---|---|---|---|---|
| | ***Early*** | ***Late*** | ***Early*** | ***Late*** | ***Early*** | ***Late*** |
| **A) Mean Solute Concentration (μM)** ± Standard Error | | | | | | |
| DOC | 55.1 ± 39 | 17.2 ± 7.9 | 375 ± 64 | 372 ± 139 | 398 ± 91.5 | 381 ± 107 |
| $NO_3^-$ | 7.13 ± 4.00 | 101.0 ± 27.8 | 1.90 ± 1.6 | 3.27 ± 2.2 | 0.79 1.46 | 2.72 ± 4.10 |
| SRP | 0.12 ± 0.06 | 0.052 ± 0.03 | 0.10 ± 0.03 | 0.06 ± 0.05 | 0.045 ± 0.03 | 0.083 ± 0.14 |
| **B) Solute Variance Collapse ($km^2$)** | | | | | | |
| DOC | 11 | 9 | 20 | 18 | 8 | 15 |
| $NO_3^-$ | 21 | 21 | 20 | 25 | 3 | 8 |
| SRP | - | - | 24 | 28 | - | - |
| **C) Mean Subcatchment Leverage ($km^2$)** ± Variance | | | | | | |
| DOC | -5.72 ± 52.5 | -4.2 ± 26.1 | -0.56 ± 2.7 | -2.1 ± 28.2 | -1.25 ± 3.3 | -3.3 ± 22.1 |
| $NO_3^-$ | 1.13 ± 35.7 | 3.1 ± 11.8 | 5.8 ± 115.6 | 7.4 ± 172.1 | -5.9 ± 98.6 | 1.7 ± 300 |
| SRP | 5.75 ± 152.5 | -2.3 ± 219.9 | -3.8 ± 42.9 | -7.2 ± 169.0 | 13.6 ± 743 | -2.3 ± 219.9 |
| **D) Spatial Stability Rank Correlation (rho)** | | | | | | |
| DOC | 0.62 | | 0.82 | | 0.12 | |
| $NO_3^-$ | 0.35 | | 0.23 | | 0.45 | |
| SRP | 0.55 | | 0.19 | | 0.03 | |

SI Table 4: Summary statistics for 2-way ANOVAs comparing mean subcatchment concentrations and leverage across catchments and seasons. Tukey's HSD reported when $p < 0.05$.

| | **Solute** | **Catchment Differences (Tukey's HSD, p < 0.05)** | **Season** | **ANOVA F value** | **df** | **p** |
|---|---|---|---|---|---|---|
| Solute Concentrations | DOC | *** (Alpine < Lake < Tundra) | * | 233 | 3,210 | < 0.001 |
| | $NO_3$ | * (Lake < Tundra = Alpine) | *** | 88.4 | 3,195 | < 0.001 |
| | SRP | ** (Alpine = Tundra < Lake) | * | 3.4 | 3,188 | 0.018 |
| Mean Subcatchment Leverage | DOC | *** Alpine = Lake > Tundra | NS | 6.9 | 3,210 | < 0.001 |
| | NO3 | *** Tundra > Alpine > Lake | * | 9.0 | 3,195 | < 0.001 |
| | SRP | *** Tundra < Alpine < Lake | ** | 11.8 | 3,188 | < 0.001 |

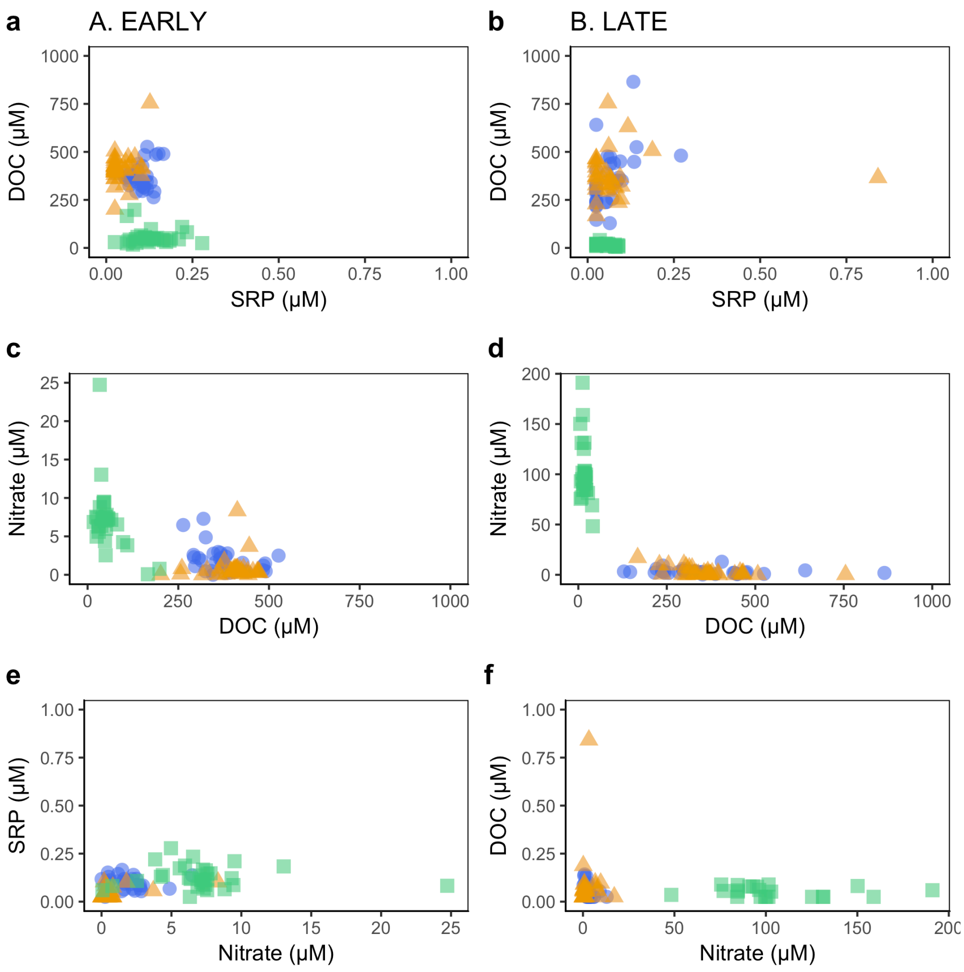


SI Figure 1: A) Early and B) Late season biogeochemical signatures comparing solutes a. nitrate ($NO_3^-$) and carbon (DOC), b. phosphorus (SRP) and $NO_3^-$, and c. DOC and SRP across our three study catchments, with Tundra (blue circles), Lake (orange triangles), and Alpine (green squares). Data for other measured solutes is provided in the supplement.

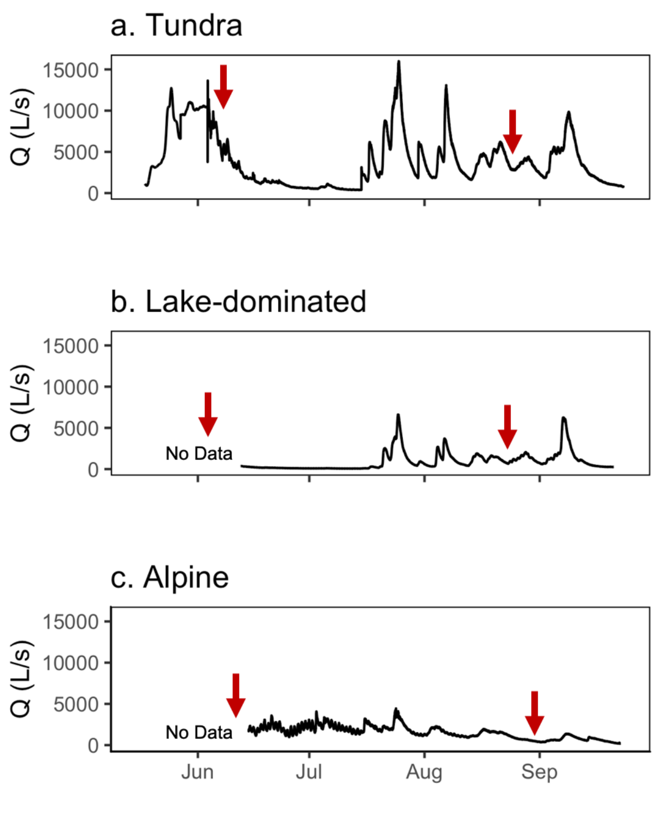


SI Figure 2: Summer 2017 hydrograph for a. Tundra, b. Lake, and c. Alpine watersheds. Estimated discharge is from the watershed outlet, using data from Hobo pressure-transducers converted to discharge using a rating curve unique to each watershed. Synoptic campaigns in each watershed are noted by red arrows.

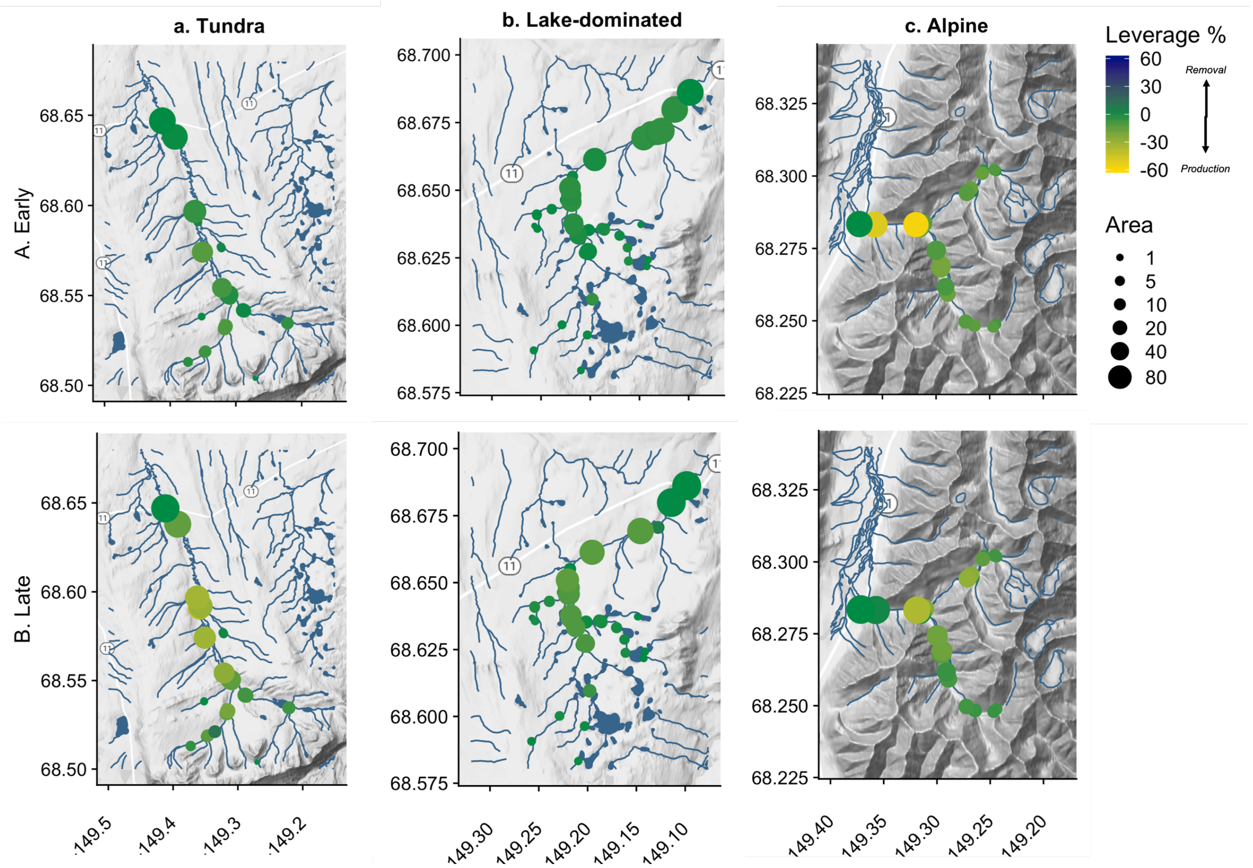


SI Figure 3: Normalized concentration (i.e., leverage) mapped for SRP from A. Early to B. Late season in the a. Tundra, b. Lake, and c. Alpine watersheds. River and lakes shown in blue. Point size indicates total subcatchment drainage area at each sampling location; smaller points indicate smaller tributaries, while the largest show main-stem or larger tributary sites. Color scale indicates leverage percent, with blue indicating lower concentrations relative to the watershed outflow and yellow noting higher concentrations than at the outflow.

## Supplemental References


1. Hobbie, J. E. & Kling, G. W. *Alaska's changing arctic : ecological consequences for tundra, streams, and lakes*.
2. Ogden, T. L. Handling results below the level of detection. *Ann. Occup. Hyg.* **54**, 255–256 (2010).
3. Killick, R. & Eckley, I. A. changepoint: An R Package for Changepoint Analysis. *J. Stat. Software3* **58**, 1–19 (2014).

4. Abbott, B. W. *et al.* Unexpected spatial stability of water chemistry in headwater stream networks. *Ecol. Lett.* **21**, 296–308 (2018).
5. Kahle, D. & Wickham, H. *ggmap: Spatial Visualization with ggplot2*.
6. Bates, D., Machler, M., Bolker, B. & Walker, S. Fitting Linear Mixed-Effects Models using lme4. *R Doc.* (2014).
